# A Novel Artificial Intelligence-Based Approach for Quantitative Assessment of Angiogenesis in the Ex Ovo CAM Model

**DOI:** 10.3390/cancers14174273

**Published:** 2022-09-01

**Authors:** Lorenz Faihs, Bardia Firouz, Paul Slezak, Cyrill Slezak, Michael Weißensteiner, Thomas Ebner, Nassim Ghaffari Tabrizi-Wizsy, Kurt Schicho, Peter Dungel

**Affiliations:** 1 Department of Oral and Maxillofacial Surgery, Medical University of Vienna, 1090 Vienna, Austria; 2 Ludwig Boltzmann Institute for Experimental and Clinical Traumatology, 1200 Vienna, Austria; 3 Department of Physics, Utah Valley University, Orem, UT 84058, USA; 4 KML Vision GmbH, 8020 Graz, Austria; 5 Otto Loewi Research Center, Department of Immunology and Pathophysiology, SFL Chicken CAM Lab, Medical University of Graz, 8036 Graz, Austria

**Keywords:** chorioallantoic membrane assay, angiogenesis, comparison of image analysis methods for angiogenesis, artificial intelligence

## Abstract

**Simple Summary:**

Angiogenesis is crucial in tissue regeneration and a relevant factor in tumor growth. Consequently, there is a demand for methods to assess angiogenesis. The chorioallantoic membrane (CAM) assay is a widely used in vivo model for the study of angiogenesis. However, there is no established gold standard to evaluate the vascularisation of the CAM, which limits the comparability of the different studies. In this manuscript, we present a novel approach to address this topic.

**Abstract:**

Angiogenesis is a highly regulated process. It promotes tissue regeneration and contributes to tumor growth. Existing therapeutic concepts interfere with different steps of angiogenesis. The quantification of the vasculature is of crucial importance for research on angiogenetic effects. The chorioallantoic membrane (CAM) assay is widely used in the study of angiogenesis. Ex ovo cultured chick embryos develop an easily accessible, highly vascularised membrane on the surface. Tumor xenografts can be incubated on this membrane enabling studies on cancer angiogenesis and other major hallmarks. However, there is no commonly accepted gold standard for the quantification of the vasculature of the CAM. We compared four widely used measurement techniques to identify the most appropriate one for the quantification of the vascular network of the CAM. The comparison of the different quantification methods suggested that the CAM assay application on the IKOSA platform is the most suitable image analysis application for the vasculature of the CAM. The new CAM application on the IKOSA platform turned out to be a reliable and feasible tool for practical use in angiogenesis research. This novel image analysis software enables a deeper exploration of various aspects of angiogenesis and might support future research on new anti-angiogenic strategies for cancer treatment.

## 1. Introduction

Angiogenesis is a critical biological process that ensures blood supply to cells. Furthermore, it is a crucial factor in tissue regeneration, as well as in pathological processes such as tumor development. New vessels are formed from pre-existing ones by means of sprouting or intussusceptive angiogenesis. Angiogenesis is a highly regulated process. The existing therapeutic concepts intervene with the different steps of angiogenesis.

The chick egg chorioallantoic membrane (CAM) assay is a widely used model to study angiogenesis. The chorioallantoic membrane is a highly vascularised membrane within fertilised and incubated chicken eggs that transports gases, metabolites, and minerals. The ex ovo CAM model offers a convenient opportunity for angiogenesis research on the easily accessible vascular network on the surface, which can easily be imaged [1,2,3]. The highly vascularized membrane offers the possibility to use CAM as a bioreactor to cultivate different tissue grafts [4]. Tumor xenografts can be transplanted onto the CAM offering an alternative in vivo model to study cancer hallmarks, such as angiogenesis [3]. Furthermore, in the gelatin sponge–chorioallantoic membrane assay, different drugs, used as antiangiogenic agents, may be tested as new treatment strategies for cancer [5].

In numerous CAM studies, different methods of quantifying vasculature have been used and described [6,7,8]. The manual count of the vessels dates back to Folkman’s research group’s first use of the CAM assay in 1974. [9]. However, manual analysis is quite time-consuming and prone to human bias. Automated and semi-automated applications enable the quantitative assessment of various vasculature morphometric and spatial parameters. The performance of the different measurement methods has to be validated. Nevertheless, the lack of a commonly accepted gold standard for quantifying the vasculature of the CAM limits the comparability of the different studies.

In this article, we present a user-friendly, standardised, and reproducible experimental design for studies on angiogenic effects with the help of artificial intelligence-based image analysis.

For this purpose, we compared widely used measurement techniques to identify the most appropriate one for the quantification of the vascular network of the CAM.

## 2. Materials and Methods

### 2.1. Ex Ovo Chorioallantoic Membrane Assay

**Figure 1:**
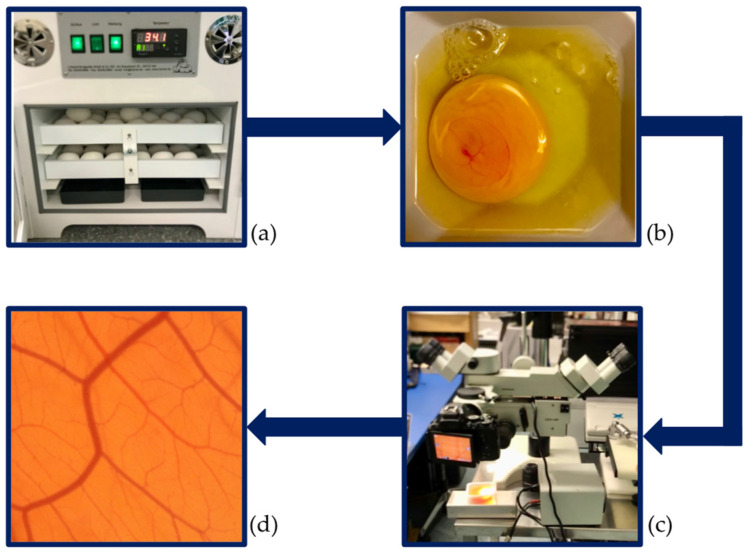
Experimental design. (**a**) Start of incubation. (**b**) Cracking of the eggs and transfer of the embryos into weight boats on day 3 of development. (**c**) Imaging of the vascular network at day 10 of development. (**d**) Image input for the analysis.

Ethics committee approval is not necessary for studies with CAM assays as chick embryos are not defined as animals until hatching [1,3]. Our study was conducted per the consensus guidelines by Schneider-Stock and Ribatti 2020 [3]. Fertilised white Lohmann chicken eggs (LSL-hatching eggs, Schropper GmbH, Gloggnitz, Austria) were incubated lying horizontally at a temperature of 37.6  °C and a constant humidity of 60% while being turned automatically for three days in an incubator (Easy 100, J.Hemel Brutgeräte, Verl, Germany). The incubator and all working areas were disinfected to prevent infection of the immunodeficient chick embryos [1]. On day 3 of incubation, all the eggs were cracked, placed into sterile weight boats, covered with a sterile square petri dish, and then put back in the incubator. On day 10 of incubation, all the CAMs were imaged, as described in subchapter two, and then euthanised. An overview of the experimental setup can be found in Figure 1.

### 2.2. Imaging

On the tenth day of development, all the chorioallantoic membranes of the surviving embryos were imaged. Imaging was performed using a light microscope (Leica M651, Leica Microsystems, Wetzlar, Germany) at tenfold magnification and a standard SLR camera (NIKON D5600, Nikon Corp, Tokyo, Japan).

### 2.3. Image Analysis

There are various methods available to analyse images of the CAM vascular network. On the one hand, there is the possibility of manual analysis. On the other hand, several applications allow an automatic or semi-automatic quantification of various vascular network parameters. Some quantification methods for CAM assays have been described; however, there is no comparison of the different image analysis methods available in the existing literature. In our present study, 100 randomly picked pictures of the vascular network of chorioallantoic membranes were analysed using each measurement technique to compare the results.

#### 2.3.1. Manual Analysis

The manual analysis of the vascular network of CAMs is very time-consuming, especially for larger sample sizes. Furthermore, the manual analysis is prone to human error and bias. We performed the manual analysis with the Software ImageJ (National Institutes of Health, Bethesda, MD, USA). It is possible to count the branching points of the vasculature with the ‘Multi-point Tool’. Furthermore, the mean vessel thickness can be evaluated by measuring a random sample of vessels with the straight-line selection tool. Our study included the mean thickness of 20 randomly picked vessels.

#### 2.3.2. ImageScope

The Software ImageScope can be used to quantify the number of pixels in an image within a specific colour range. Marinaccio et al. described how to assess the vascularisation of the CAM with the ‘Positive Pixel Count v9’ tool embedded in ImageScope. The algorithm yields the number of strong positive pixels in %, which is a morphometric value for the quantification of the surface of the vasculature [7].

#### 2.3.3. AngioTool

Another widely used application to analyse vascular networks is the freely available, open-source software AngioTool. It collects data on morphological and spatial parameters, such as the total area of the vascular network, the number of vessels, vessel density, length, and lacunarity [10].

#### 2.3.4. IKOSA

Recent developments in artificial intelligence (AI) have opened new possibilities for image analysis, especially in life sciences. The IKOSA platform [11] provides various analysis tools whereby one of them, called “CAM Assay”, facilitates AI-based analysis of vascular networks on CAM images. With IKOSA, quantitative information such as the vessel area and length can be extracted. The underlying image analysis model is based on a fully convolutional neural network [12], which uses an InceptionResNetv2 [13] as an encoder and a U-Net [14] based structure as a decoder path. The model was trained in 2 stages: The first training stage included manually annotated blood vessels of (i) 322 retinal fundus images. Each image had a size of 1024 × 1024 pixels. During training, random image patches of size 256 × 256 were cropped, normalised, and augmented by various commonly used methods such as random horizontal and vertical flipping. In the second training stage, the previously trained model was additionally trained on the target (ii) CAM dataset, consisting of 8 training images with each being 6000 × 4000 pixels. To provide a reasonably sized receptive field to the network, the resolution of each image was reduced to 1536 × 1024 pixels. Again, image patches of size 256 × 256 pixels were cropped during training, using the same augmentation methods as for the previous training. Since the trained model of the first stage was used as a basis to be re-trained on a (slightly) different task, this can also be referred to as transfer learning [15,16]. Especially for training on a very small number of training images, the principle of transfer learning is often superior to training the same model from scratch, which is also concluded by Ghafoorian et al. [16]. When applying the final model to CAM images, we obtain a segmentation map in terms of a binary mask representing the vessel area at pixel level. Based on that, prediction postprocessing and statistical analysis finally yield different output parameters such as the total vessel area, the total vessel length, the mean vessel thickness, and the number of vessel branching points.

**Figure 2:**
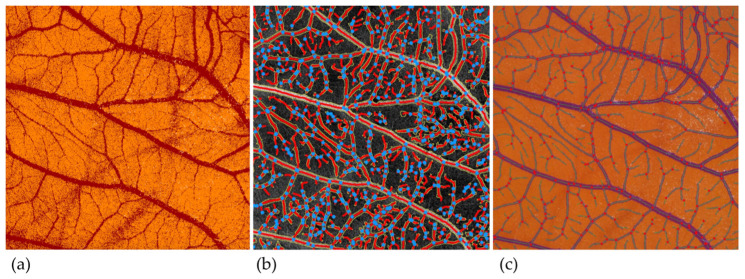
Segmentation masks with different image analysis applications. (**a**) ImageScope. (**b**) AngioTool. (**c**) IKOSA CAM Assay application.

An overview of the segmentation masks of the different image analysis methods can be found in Figure 2.

#### 2.3.5. Statistical Analysis

All statistical analyses were performed with SPSS for Mac, Version 27 (IBM, Armonk, NY, USA). To compare the different image analysis methods, we created Bland–Altman plots to visualise and describe the advantages and disadvantages of each application. Furthermore, we conducted paired sample t-tests with branching points outputs of the different measurement methods. Comparing the means was not reasonable for the other parameters as the results might have differed because of the different input parameters.

## 3. Results

We examined the advantages and disadvantages of several widely used image analysis methods for vascular networks. Not all measurement techniques provide the same morphological and specific parameters. Some image analysis methods cannot be compared with each other as some of the applications yield no corresponding parameters. The Bland–Altman plots show the concordance of the measurements. The green line depicts the mean difference. The two red lines represent the level of agreement (average difference ± 1.96 × standard deviation of the difference).

### 3.1. Branching Points

The number of branching points is a well-suited parameter to compare different methods for quantifying vascular networks. It is an absolute number that does not depend on the selected input parameters. Branching points are a main parameter for assessing angiogenesis as new vascular branches develop by sprouting or intussusception from pre-existing vessels [2].

**Figure 3:**
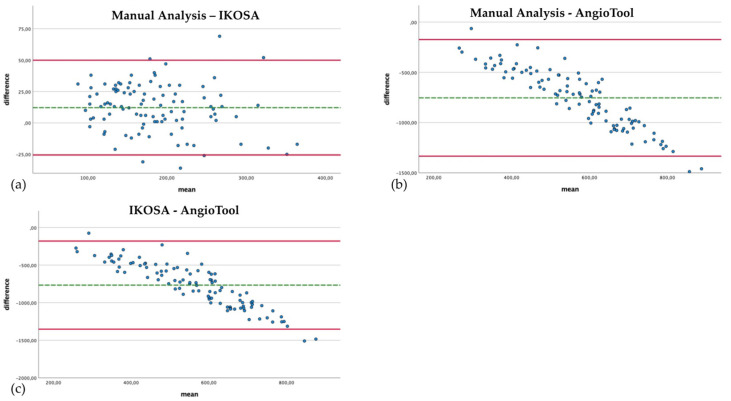
Bland–Altman plots with a comparison of the branching points of the vascular networks quantified with the different image analysis methods. (**a**) Manual analysis, IKOSA CAM Assay application. (**b**) Manual analysis, AngioTool. (**c**) IKOSA CAM Assay application, AngioTool.

The manual count of the branching points is very time intensive. However, if carried out conscientiously, it is a reliable method to quantify the number of branching points. Figure 3 compares the quantification of branching points with the different image analysis methods.

The IKOSA CAM Assay application results show the smallest difference compared to the manual analysis (mean difference 12.18, SD 19.26). When manually counted, significantly (*p* < 0.001) more branching points could be detected (mean 190.61) than with the application on the IKOSA platform (mean 178.43). The quantification of branching points with AngioTool (mean 944.26) delivers a significantly (*p* < 0.001) higher number of vascular branches than the manual counting method (mean 190.61) (mean difference −753.65, SD 296.18). The difference between the measurement methods is more apparent for images with a higher number of branching points. This effect can also be observed in comparing the IKOSA CAM Assay application with AngioTool (mean difference −765.83, SD 298.93). Here, a significantly (*p* < 0.001) higher number of vascular branches is also detected with AngioTool (mean 944.26) than with the application on IKOSA (mean 178.43).

The validation of the new CAM application on IKOSA is mainly based on the number of branching points as parameters for the vasculature. Branching points can clearly be identified in the course of the manual analysis. They are a reproducible parameter.

Moreover, a visual validation has been performed by evaluating the segmentation masks after the image analysis. Visual analysis of the segmentation masks is reliable as human visual perception is highly sensitive to detecting incongruences.

### 3.2. Total Length

Another critical process during angiogenesis is vessel elongation [17]. The appearance of new branches and pre-existing vessels’ elongation influence the vascular network’s total length. The total length of the vessels in an image can be determined with AngioTool and the new application on IKOSA.

**Figure 4:**
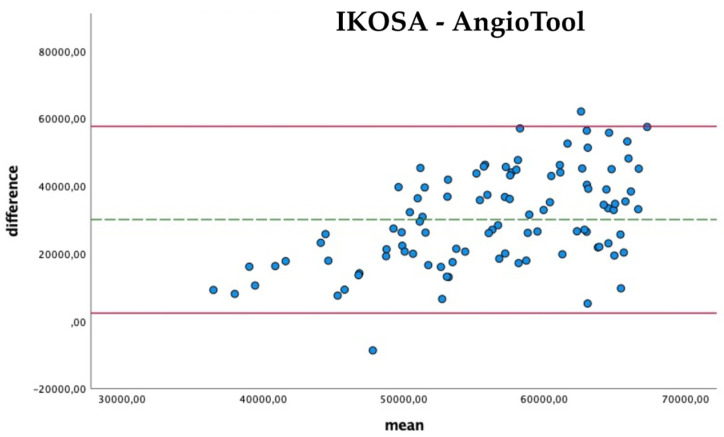
Bland–Altman Plots with a comparison of the total vasculature length measured with the IKOSA CAM Assay application and AngioTool.

The comparison of the results (Figure 4) shows that the difference in the measurements with the two applications becomes apparent in pictures with a higher total vascular length (mean difference 30,048.67 Px, SD 14,084.06). The results of AngioTool (mean 71,311.21 Px) are higher than those collected with the IKOSA CAM Assay application (mean 41,262.54 Px).

### 3.3. Total Area

The total area quantifies the surface of all vessels in an image. The vascular area increases as the vascular network grows. However, it also depends on the thickness of the blood vessels. New vascular sprouts appearing during angiogenesis are thin and might not impact the total vascular area as much as other parameters. Furthermore, the input parameters of the image analysis application differ; consequently, the results might diverge. There is no gold standard for the quantification of the vascular area. Since the measurement of the surface of all vessels cannot be determined using manual analysis, only a comparison of the quantification software outputs is possible.

**Figure 5:**
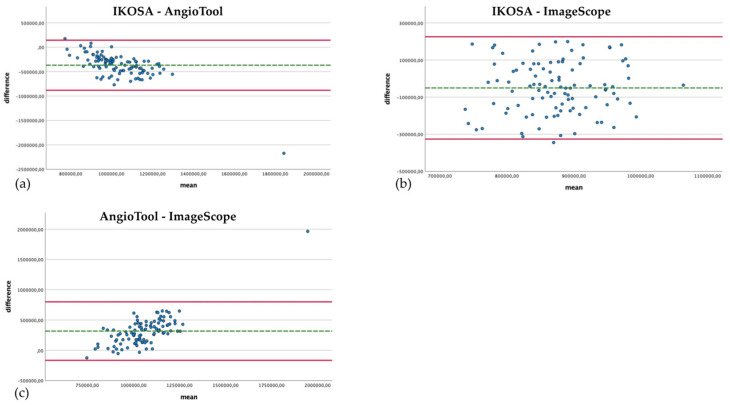
Bland–Altman plots with a comparison of the total area of the vasculature measured with different image analysis methods. (**a**) IKOSA CAM Assay application, AngioTool. (**b**) IKOSA CAM Assay application, ImageScope. (**c**) AngioTool, ImageScope.

The comparison of the total area quantified with the different image analysis applications is depicted in Figure 5. The difference in the results for the vascular area from AngioTool (mean 1,217,033.23 Px^2^) and ImageScope (mean 899,284.93 Px^2^) seems to be the smallest, with a greater difference for images with a larger vascular area (mean difference 317,748.3 Px^2^, SD 246,416.687). This divergence can also be seen in the comparison of the application on IKOSA (mean 848,942.21 Px^2^) and AngioTool (mean 1,217,033.23 Px^2^), where images with a larger surface area of blood vessels show higher differences in the results (mean difference −368,091.02 Px^2^, SD 261,717.82). The measured vascular area with the IKOSA CAM Assay application and ImageScope has the biggest difference (mean difference −50,342.72 Px^2^, SD 140,711.099). ImageScope (mean 899,284.93 Px^2^) measures a higher vascular area than the application on IKOSA (mean 848,942.21 Px^2^).

### 3.4. Mean Thickness

Changes in the mean thickness of the vasculature are related to angiogenic processes. New thin sprouts develop, leading to a decrease in the mean diameter of the vascular network. The mean thickness can be assessed using manual measurement of the diameter of a random sample of vessels or with the IKOSA CAM Assay application.

**Figure 6:**
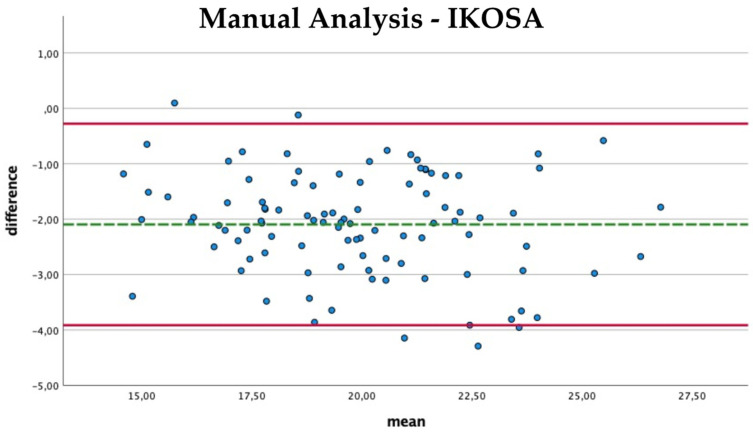
Bland–Altman plot with the comparison of the mean thickness of the vasculature measured manually and with the IKOSA CAM Assay application.

The comparison of the mean thickness of the vascular network using manual analysis and with the application on IKOSA (Figure 6) shows a slight difference in the results (mean difference −2.09 Px, SD 0.93). Furthermore, no trend for measurement errors in pictures with high or low thickness values can be found. The IKOSA CAM Assay application (mean 20.92 Px) tends to measure a higher mean thickness than the manual measurement method (mean 18.8 Px).

## 4. Discussion

In the present study, we compared the widely used image analysis methods for the vasculature of the CAM. We presented a new AI-based approach for quantifying the vasculature of the CAM and evaluated the sensitivity and practical use.

The new AI-based image analysis CAM Assay application on the IKOSA platform is a convenient and accurate method to quantify the vascular network of the chorioallantoic membrane. Our results show that this application is the most precise method for analysing the branching points and the mean vascular thickness compared to the standard, manual count. In addition, it provides the advantage of evaluating further vascular parameters, which cannot be assessed manually.

**Figure 7:**
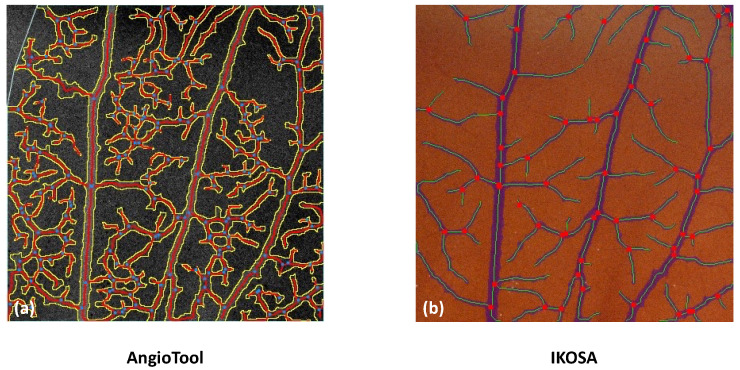
Comparison of the image analysis with (**a**) AngioTool and (**b**) the IKOSA CAM Assay application.

The data analysis with AngioTool revealed higher values for all parameters than all other analysis methods. This could indicate a measurement error whereby vessels are falsely detected, as shown in Figure 7.

**Figure 8:**
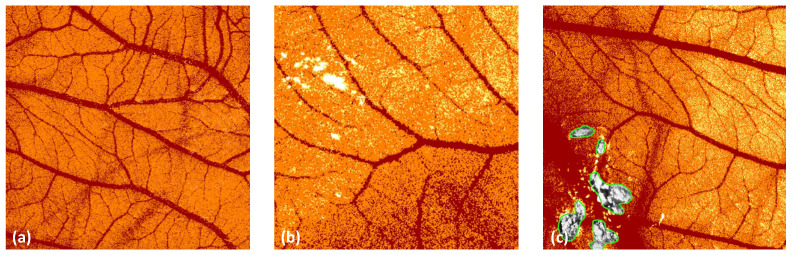
Image analysis with ImageScope. (**a**) Artefacts related to specific background structures (here: deeper blood vessels). (**b**) Light artefacts due to light reflections. (**c**) Exclusion of areas with the ‘negative pen tool’.

ImageScope delivers a rough estimation of the surface of the vessels in an image. Other spatial or morphometric parameters cannot be evaluated. The algorithm ‘Positive Pixel Count v9’ does not directly detect the vessels but detects pixels in a specific colour range. Image artefacts, such as light reflections, may distort the results, as depicted in Figure 8. These artefacts can be excluded from the analysis with the negative pen tool embedded in ImageScope. Reviewing the images prior to the analysis to exclude these artefacts is time-consuming and might bias the results.

The possibility of automated image analysis enables studies with high sample sizes and consequently a higher predictive power. Furthermore, it permits the quantification of various morphometric and spatial parameters. Treatments and drugs influence different vasculature parameters [8]. While some of these parameters cannot be quantified manually, image analysis software enables a deeper exploration of various aspects of angiogenesis.

We believe our work will shed light on new directions for using the CAM Assay in broad areas of medical research.

## 5. Conclusions

In conclusion, we demonstrated the practical use of a new AI-based automated quantification method for angiogenesis in CAM. Compared to the widely used image analysis methods, the IKOSA platform turned out to be a reliable and feasible tool for practical use in research on angiogenesis. This new image analysis software enables a deeper exploration of various aspects of angiogenesis and might support future research. The CAM Assay provides an alternative in vivo model for angiogenesis in tumor development. In the gelatin sponge–chorioallantoic membrane assay, different drugs, such as antiangiogenic agents may be tested. The CAM model is an inexpensive and easy-to-use model to study new anti-angiogenic strategies for cancer treatment. Reliable quantification of vascularisation is indispensable for meaningful research.

## Data Availability

Not applicable.
